# Bevacizumab versus Ranibizumab on As-Needed Treatment Regimen for Neovascular Age-Related Macular Degeneration in Turkish Patients

**DOI:** 10.1155/2013/151027

**Published:** 2013-08-29

**Authors:** Abdullah Ozkaya, Zeynep Alkin, Yalcin Karakucuk, Dilek Yasa, Ahmet Taylan Yazici, Ahmet Demirok

**Affiliations:** ^1^Beyoglu Eye Training and Research Hospital, Bereketzade Camii Sok., Kuledibi, Beyoglu, 34421 Istanbul, Turkey; ^2^Department of Ophthalmology, Medeniyet University, Istanbul, Turkey

## Abstract

*Purpose*. To compare the efficacy of intravitreal bevacizumab versus ranibizumab in the treatment of neovascular age-related macular degeneration (nAMD). *Methods*. Retrospective, comparative study. The newly diagnosed nAMD patients who were treated with intravitreal bevacizumab or ranibizumab on an as-needed treatment regimen were included in the study. Main outcome measures were the change in best corrected visual acuity (BCVA), and central retinal thickness (CRT). Secondary outcome measures were the number of injections, and complications. *Results*. A total of 154 patients were included in the study. Bevacizumab group consisted of 79 patients, and ranibizumab group consisted of 74 patients. Mean follow-up time was 18.9 months, and 18.3 months in the bevacizumab and ranibizumab groups, respectively. There was not a significant difference between the two groups regarding the change in BCVA and CRT at all time points (*P* > 0.05 for all). The mean number of injections at month 12 was 4.8 and 4.7 in bevacizumab and ranibizumab groups, respectively (*P* > 0.05). No serious complications were detected in any of the groups. *Conclusion*. Both of the bevacizumab and ranibizumab found to be effective in the treatment of nAMD in regards of functional and anatomical outcomes with similar number of treatments and similar side effects.

## 1. Introduction

Neovascular age-related macular degeneration (nAMD) is a leading cause of visual loss among elderly population [1, 2]. Before the introduction of intravitreal antivascular endothelial growth factor (anti-VEGF) agents for the treatment of nAMD, only prevention from visual acuity loss might have been achieved in a limited number of patients with different treatment options like laser photocoagulation, photodynamic therapy, and vitreoretinal surgery [3–9]. Intravitreal treatments with bevacizumab (full length antibody against VEGF-A) and ranibizumab (Fab part of antibody against VEGF-A) have led the majority of the patients to prevent the baseline visual acuity (VA) and even to achieve visual improvement in some of the patients [10, 11]. The multicenter studies with ranibizumab, like MARINA, ANCHOR, PRONTO, and EXCITE, and the comparative study of ranibizumab and bevacizumab, the CATT study, showed that ranibizumab and bevacizumab were effective to prevent VA loss up to 95% of the patients and is effective to make an improvement in VA up to 40% of the patients [11–15]. Today, the CATT trial answered the questions about the efficacy of bevacizumab versus ranibizumab and showed that both drugs had similar effects in the treatment of nAMD. However, the safety of the drugs still remains unclear, and there is an ongoing debate about this issue.

In this study, we aimed to compare the functional and anatomical outcomes of bevacizumab therapy to ranibizumab therapy on as-needed treatment regimen for nAMD in Turkish patients.

## 2. Material and Methods

The clinical data of the patients who were treated with either bevacizumab or ranibizumab between 2009 and 2011 were retrospectively evaluated for this study. Inclusion criteria were age of 50 years or more, newly diagnosed nAMD, no treatments received other than bevacizumab alone or ranibizumab alone, and a minimum follow-up period of 12 months. Patients were not included if they had any of the following criteria: retinal diseases other than nAMD, previous intravitreal injections, or a history of photodynamic therapy or laser photocoagulation. The tenets of the Declaration of Helsinki were followed throughout the study, and written informed consent was obtained from all patients before the treatments were started.

### 2.1. Data

Data collected from the patients' records included age, gender, type of choroidal neovascularization (CNV) (predominantly or minimally classic or occult), BCVA, and central retinal thickness (CRT) before treatment, at months 3, 6, 9, and 12 during treatment, and at the most recent follow-up examination. Cumulative numbers of injections were recorded for each patient.

### 2.2. Examinations

All patients underwent a standardized examination including measurement of BCVA via the Early Treatment Diabetic Retinopathy Study (ETDRS) chart at 4 meters, slit-lamp biomicroscopy and fundus examination and measurement of intraocular pressure (IOP) via applanation tonometry. Fundus photography, fluorescein angiography (HRA-2, Heidelberg Engineering, Heidelberg, Germany), and optical coherence tomography (OCT) imaging (Stratus OCT TM, Carl Zeiss Meditec Inc., Dublin, CA, USA) were performed before treatment. All examinations were repeated monthly, except fluorescein angiography, which was repeated only when the cause of visual acuity deterioration could not be clarified with the clinical examination and other imaging methods. OCT was used for detecting subretinal fluid and measurement of CRT, the latter being defined as the mean thickness of the neurosensory retina in the central 1 mm diameter region, computed via OCT mapping software provided with the device.

### 2.3. Injection Method

All injections were performed under sterile conditions after topical anesthesia, and 10% povidone-iodine scrub (Betadine, Purdue Pharma, Stamford, CT, USA) was used on the lids and lashes, and then 5% povidone-iodine was administered on the conjunctival sac. Intravitreal bevacizumab (Avastin, Genentech, South San Francisco, CA, USA) or ranibizumab (Lucentis, Novartis, Basel, Switzerland) was injected with a 30-gauge needle through the pars plana at 3.5 mm to 4 mm posterior to the limbus. Patients were then instructed to consult the hospital if they experienced decreased vision, eye pain, or any new symptoms.

### 2.4. Treatment Schedule

For the first three months of treatment, all patients received monthly doses of bevacizumab, or ranibizumab (1.25 mg/0.05 mL for bevacizumab, 0.5 mg/0.05 mL for ranibizumab). The patients were then examined monthly and were retreated if they met any of the following criteria:

- visual loss of 1 or more lines,
- newly developed macular hemorrhage,
- evidence of CNV enlargement on examination or fluorescein angiography,
- any amount of persistent subretinal fluid one month after an injection.

### 2.5. Data Analysis

Visual acuity was converted to logarithm of Minimum Angle of Resolution (logMAR) for statistical analysis. The changes in BCVA and CRT over time were analyzed with paired sample *t*-test. Chi-square test was used to compare nominal parameters between the groups, and independent sample *t*-test was used for continuous parameters. The statistical analysis was performed using SPSS version (Version 15.0, SPSS Inc., Chicago, IL, USA). A *P* value of less than 0.05 was considered to be statistically significant.

## 3. Results

A total of 154 patients met the inclusion criteria for the study. The bevacizumab group consisted of 79 patients, and the ranibizumab group consisted of 74 patients. The general characteristics of the patients were similar between the two groups (Table 1). 

**Figure 1:**
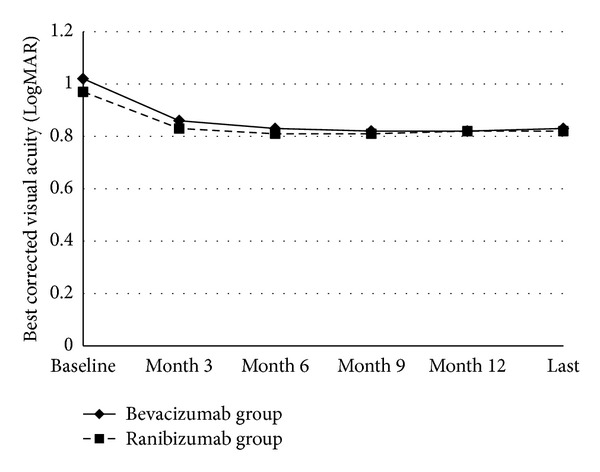
The changes in mean visual acuity in the bevacizumab group and ranibizumab groups. The graph shows the mean LogMAR visual acuity levels from baseline to the most recent followup. The change in mean best corrected visual acuity from the baseline to months 3, 6, 9, 12, and the last visit (Last) was not statistically different between the two groups (*P* > 0.05 for all).

The mean BCVA of the patients in the bevacizumab group at baseline, months 3, 6, 9, 12, and at the most recent followup was 1.02 ± 0.46 LogMAR (range 0.1–2.1 LogMAR), 0.86 ± 0.46 LogMAR (range 0.2–2.1 LogMAR), 0.83 ± 0.43 LogMAR (range 0.2–2.1 LogMAR), 0.82 ± 0.43 LogMAR (range 0.2–2.1 LogMAR), 0.82 ± 0.42 (range 0.2–2.1 LogMAR), and 0.83 ± 0.43 (range 0.2–2.1 LogMAR), respectively. The mean BCVA of the patients in the ranibizumab group at baseline, months 3, 6, 9, 12, and at the most recent followup was 0.97 ± 0.46 LogMAR (range 0.1–2.1 LogMAR), 0.83 ± 0.47 LogMAR (range 0.1–1.8 LogMAR), 0.81 ± 0.47 LogMAR (range 0.0–1.8 LogMAR), 0.81 ± 0.42 LogMAR (range 0.1–1.8 LogMAR), 0.82 ± 0.43 (range 0.0–1.8 LogMAR), and 0.82 ± 0.47 (range 0.0–2.1 LogMAR), respectively (Figure 1). The change in mean BCVA from baseline to months 3, 6, 9, 12, and the most recent followup was statistically better in both of the groups (*P* < 0.05, for all). However, there was not a statistically significant difference between the two groups in regards of change in BCVA at all of the time points (*P* = 0.7 for month 3, *P* = 0.6 for month 6, *P* = 0.5 for month 9, *P* = 0.5 for month 12, and *P* = 0.6 for the most recent followup) (Table 2).

At the most recent followup, 34 of the 79 patients (43.0%) in the bevacizumab group and 30 of the 74 patients (40.5%) in the ranibizumab group gained VA ≥ 3 lines (*P* = 0.4). Sixty-eight of the 79 patients (86.1%) in the bevacizumab group and 64 of the 74 patients (86.5%) in the ranibizumab group had stable or improved vision (loss of < 3 lines, remained stable, or gained ≥ 1 lines) (*P* = 0.5). Eleven of the 79 patients (13.9%) in the bevacizumab group and 10 of the 74 patients (13.5%) in the ranibizumab group had VA loss ≥ 3 lines (*P* = 0.5). 

**Figure 2:**
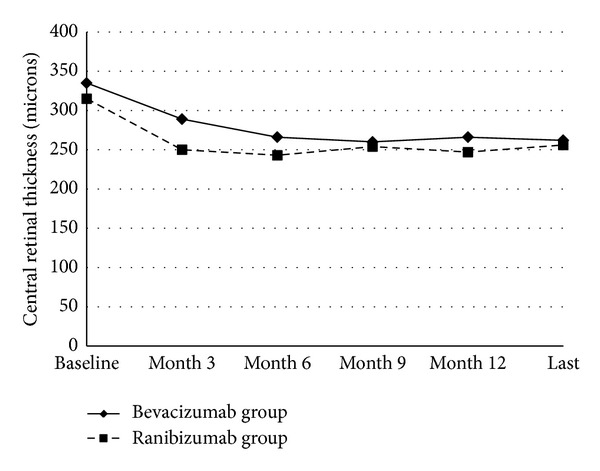
The changes in mean central retinal thickness in the bevacizumab group and ranibizumab group. The graph shows the mean central retinal thickness levels from baseline to the most recent followup. The change in mean central retinal thickness from the baseline to months 3, 6, 9, 12, and the last visit (Last) was not statistically different between the two groups (*P* > 0.05 for all).

The mean CRT in the bevacizumab group at baseline, and at months 3, 6, 9, and 12, and at the most recent followup was 335 ± 120 *μ*m (range 180–758 *μ*m), 289 ± 118 *μ*m (range 106–796 *μ*m), 266 ± 79 *μ*m (range 140–558 *μ*m), 260 ± 93 *μ*m (range 148–713 *μ*m), 266 ± 100 (range 150–789 *μ*m), and 262 ± 124 (range 103–979 *μ*m), respectively. The mean CRT in the ranibizumab group at the baseline, and at months 3, 6, 9, and 12, and at the most recent followup was 315 ± 88 (range 139–681 *μ*m), 250 ± 68 *μ*m (range 133–487 *μ*m), 243 ± 63 *μ*m (range 135–410 *μ*m), 254 ± 72 *μ*m (range 135–540 *μ*m), 247 ± 60 (range 140–415 *μ*m), and 256 ± 73 (range 130–483 *μ*m), respectively (Figure 2). The mean CRT at months 3, 6, 9, and 12, and at the most recent followup was statistically different compared to baseline in both of the groups (*P* < 0.05 for all). However, the change in mean CRT from the baseline to months 3, 6, 9, and 12, and at the most recent follow-up was not statistically different between the two groups (*P* = 0.2, *P* = 0.8, *P* = 0.3, *P* = 0.9, and *P* = 0.2, resp.) (Table 3).

The mean number of injections at month 12 was 4.8 ± 1.2 (range 3–7) in the bevacizumab group and 4.7 ± 1.4 (range 3–8) in the ranibizumab group, and the difference was not statistically significant between the two groups (*P* = 0.8). The mean injection numbers were summarized in Table 4.

The mean baseline intraocular pressure (IOP) was 15.5 ± 2.3 mm Hg (range 9–19 mm Hg) and 15.4 ± 1.7 (range 12–20 mm Hg) in the bevacizumab and ranibizumab groups, respectively (*P* = 0.7). The mean IOP at the most recent followup was 15.3 ± 2.4 mm Hg (range 10–22 mm Hg) and 15.6 ± 2.1 (range 11–22 mm Hg), in the bevacizumab and ranibizumab groups, respectively (*P* = 0.4). 

No serious ocular or systemic complications were observed in any of the patients. Only mild complications like punctate keratitis, subconjunctival hemorrhage, and transient mild anterior uveitis were detected (Table 5). 

## 4. Discussion

Functional and anatomical outcomes of nAMD patients treated with bevacizumab and ranibizumab in a population of Turkish patients were reported in this study. In both of the groups, the mean BCVA and CRT were significantly better than that in baseline at every time point. The two groups were parallel to each other with respect to change in BCVA and CRT, injection numbers, and complications. 

In previous clinical studies, these two drugs, bevacizumab or ranibizumab, have been found to provide similar improvements in visual and anatomical outcomes to our study [13, 16–20]. 

A short term efficacy and safety study by Landa et al. [16] reported that bevacizumab and ranibizumab treatments resulted in similar improvements in visual and anatomical outcomes one month after an intravitreal injection in patients with nAMD. They also stated that bevacizumab was as safe as ranibizumab. 

In a retrospective study by Feng et al. [17], 371 nAMD patients who were treated with intravitreal bevacizumab or ranibizumab on an as-needed treatment regimen were evaluated. They reported that, after a median follow-up time of 12 months, 24.5% of patients in the bevacizumab group and 25.8% of patients in the ranibizumab group had a gain of 15 or more letters; in addition, 79.5% of the patients in the bevacizumab group, and 84.9% of the patients in the ranibizumab group lost fewer than 15 letters in visual acuity. They stated that there was not a statistical difference in regards of visual acuity changes between the two groups. The results of our study are parallel to the study by Feng et al. We also did not find any difference in visual acuity change at different time points between the bevacizumab and ranibizumab groups. And the rate of the patients who were stable in regards of visual acuity results was very similar to the study by Feng et al.

Fong et al. [18] reported that bevacizumab and ranibizumab were both effective in stabilizing visual acuity in nAMD patients after 12 months of followup. In the aforementioned study, it was reported that visual acuity outcomes were similar between the two drugs; 27.3% of the patients in the bevacizumab and 20.2% of the patients in the ranibizumab group were reported to have an improvement of three or more lines in visual acuity, and the difference between the groups was not found to be statistically significant. The mean injection numbers at month 12 were 4.4 in the bevacizumab group and 6.2 in the ranibizumab group which were statistically different between the groups. The mean injection numbers at month 12 were similar to our study; however, we did not find a difference between the two groups.

In a retrospective study concerning the efficacy of bevacizumab and ranibizumab in nAMD by Bellerive et al. [19], the mean visual acuity improvement at month 12 was greater in the ranibizumab group than the bevacizumab group. This trend attributed to the variability of the lesion compositions of the groups. They stated that the proportion of occult lesions were greater in the bevacizumab group than the ranibizumab group and postulated that this trend might be explained by this variability. Also the proportion of the patients who lost fewer than 0.3 LogMAR was reported to be 83% in the bevacizumab group and 92 in the ranibizumab group, with a mean number of injections of 4.7 in the bevacizumab group and 4.9 in the ranibizumab group at month 12. The visual acuity outcomes and the mean injection numbers of the study were comparable with our study. However, the visual and anatomical outcomes were similar between the groups in our study.

The CATT study was designed as a multicenter, noninferiority trial to compare the efficacy and safety of bevacizumab and ranibizumab. In the study, both monthly and as-needed treatment regimens were evaluated within and between the two drugs. At first year, it was reported that there was not a significant difference between the two drugs in regards of visual acuity and anatomical outcomes and the mean injection numbers. At month 12, as-needed bevacizumab was found to be equivalent to as-needed ranibizumab with a gain of 5.9 ETDRS letters and 6.8 ETDRS letters in visual acuity, respectively. At month 12, the eyes treated with as-needed bevacizumab and ranibizumab showed a significant decrease in CRT, and the difference was not statistically significant between the bevacizumab groups and ranibizumab groups. The mean injection numbers of as-needed bevacizumab group and as-needed ranibizumab group were 7.7 and 6.9 at month 12, respectively. The two-year results of the study were similar to the first-year results; however the mean gain in visual acuity was found to be greater in monthly groups than as-needed groups [20]. The study also addressed the safety issue and adverse events between the two drugs. At 2 years, it was reported that 6.1% of the patients in the bevacizumab group and 5.3% of the patients in the ranibizumab group had died. The proportion of the patients with atherothrombotic events and venous thrombotic events were similar between the two groups. The rate of serious adverse events was found to be greater in the bevacizumab groups than the ranibizumab groups with a cumulative risk ratio of 1.30; however, this difference was not found to be statistically significant. In addition, the studies which addressed the safety issues of bevacizumab and ranibizumab showed that this issue still remains as a debate [21–23].

Limitation of the present study includes the relatively small numbers of patients. Strengths of the study include the fact that patients in the bevacizumab and ranibizumab groups had received no other forms of treatment and similar baseline characteristics of the two groups.

In summary, in the present study we found that 86.1% of the patients in the bevacizumab group and 86.5% of the patients in the ranibizumab group had a stable or improved visual acuity after a mean follow-up time of approximately 18 months. The visual and anatomical outcomes and the number of injections were similar between the two drugs. No patients had suffered from a serious adverse event during the entire followup. The patients with nAMD treated with as-needed bevacizumab or as-needed ranibizumab showed significant improvements in visual acuity throughout at least 1.5 years compared to baseline visual acuity. 

## Figures and Tables

**Table 1:** General characteristics of the patients.

	Bevacizumab group	Ranibizumab group	*P* value
Mean age, years	71.4 ± 8.2	73.8 ± 7.2	0.1
Gender (male/female)	36/43	42/32	0.2
Laterality (right/left eye)	38/41	34/40	0.8
Follow-up time (months)	18.9 ± 4.5	18.3 ± 4.7	0.4
Lens status (phakic/pseudophakic)	59/19	47/27	0.1
CNV type (classic/occult)	23/56	20/54	0.8

CNV: choroidal neovascularization.

**Table 2:** The change in mean best corrected visual acuity from baseline to different time points.

Change in BCVA (LogMAR line)	Month 3	Month 6	Month 9	Month 12	Last followup
Bevacizumab group	1.6	1.9	1.9	1.9	1.8
Ranibizumab group	1.4	1.5	1.5	1.5	1.5
*P* value	0.7	0.6	0.5	0.5	0.6

BCVA: best corrected visual acuity; LogMAR: logarithm of the minimal angle of resolution.

**Table 3:** The change in mean central retinal thickness from baseline to different time points.

Change in CRT (microns)	Month 3	Month 6	Month 9	Month 12	Last followup
Bevacizumab group	45	69	74	69	72
Ranibizumab group	64	71	60	67	58
*P* value	0.2	0.8	0.3	0.9	0.2

CRT: central retinal thickness, LogMAR: logarithm of the minimal angle of resolution.

**Table 4:** Mean number of injections.

	Bevacizumab group	Ranibizumab group	*P* value
First year	4.8 ± 1.2 (range 3–7)	4.7 ± 1.4 (range 3–8)	0.8
Second year	0.9 ± 1.2 (range 0–5)	1.3 ± 1.4 (range 0–4)	0.1
Total	5.7 ± 2.0 (range 3–11)	6.0 ± 2.3 (range 3–12)	0.3

**Table 5:** Complications.

	Bevacizumab group (*n* = 79 patients)	Ranibizumab group (*n* = 74 patients)	*P* value
Punctuate keratitis	10 (12.6%)	11 (14.8%)	0.7
Subconjunctival Hemorrhage	7 (8.8%)	8 (10.8%)	0.5
Mild transient Anterior uveitis	5 (6.3%)	2 (2.7%)	0.1
